# Thin Film Composite Mixed-Matrix Membranes Based on Matrimid and Zeolitic Imidazolate Frameworks for CO_2_/N_2_ Separation Performance

**DOI:** 10.1021/acs.iecr.4c03086

**Published:** 2024-11-11

**Authors:** Elsa Lasseuguette, Maria-Chiara Ferrari

**Affiliations:** School of Engineering, University of Edinburgh, Robert Stevenson Rd., Edinburgh EH9 3FB, United Kingdom

## Abstract

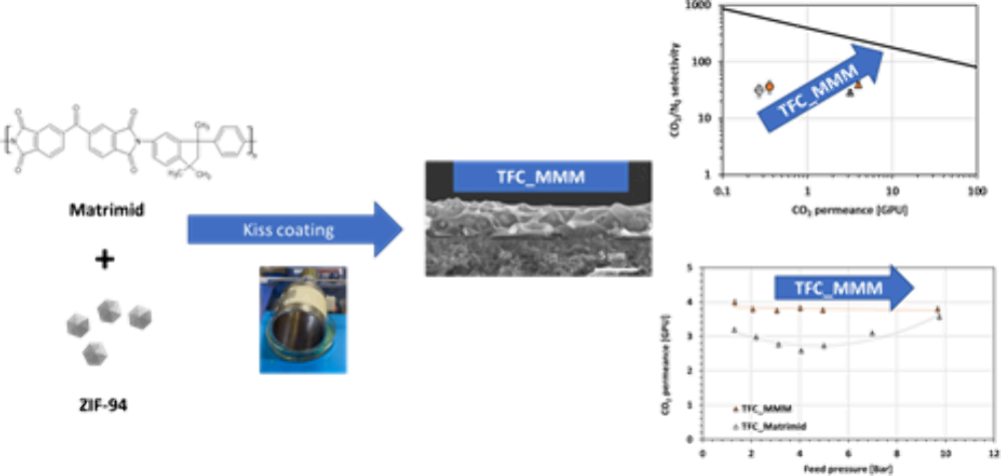

Membrane-based gas separation processes are a technology in continuous evolution. Various types of polymer membranes have been developed, many exhibiting high CO_2_ permeability and selective properties over competing gases such as N_2_ and CH_4_. In order to be competitive, membranes must be less-expensive, more stable, and more efficient, and their production must be scalable. One solution is to develop thin-film composites with mixed-matrix membranes (TFC_MMM) that have the potential to boost productivity while maintaining low costs. In this work, TFC_MMMs containing Matrimid mixed with 12 wt % ZIF-94 were prepared by kiss coating on a porous support. The SEM analysis showed that defect-free membranes with a 3 μm selective layer have been obtained. At 1 bar, the addition of ZIF resulted in improved the separation performance for the CO_2_/N_2_ pair, with CO_2_ permeance of 4 GPU and CO_2_/N_2_ selectivity of 40, surpassing neat TFC-Matrimid (CO_2_ permeance ≈ 3 GPU, CO_2_/N_2_ selectivity ≈ 29). The use of ZIF-94 also had a stabilizing effect on the membranes against CO_2_ plasticization at high pressure.

## 1 Introduction

Anthropogenic emissions to the atmosphere have caused a rapid rise in atmospheric levels of CO_2_ to values that are expected to cause dangerous extremes in climate change and sea level rise.^1^ To mitigate global CO_2_ emission, carbon capture and storage (CCS) has been proposed for current carbon-intensive industrial sectors with deployment underway. Several approaches are being developed for CO_2_ capture, including absorption by solutions of amines or alcohols, porous solid adsorption, and membrane separation.^2^ Among these, membrane-based gas separation possesses the significant advantages of small footprint and considerable energy savings. However, in order to be competitive with the traditional CO_2_ capture process, membranes should be less expensive, more stable, more efficient, i.e., with a high productivity and selectivity, and their production should be scalable.^3^ In order to increase productivity, membranes with higher permeances are needed. Decreasing the membrane thickness results in higher permeance; however, this must be carefully balanced, maintaining mechanical stability and integrity. Thus, a feasible solution to obtain very thin defect-free membranes is to develop a thin-film composite (TFC) material, consisting of a thin selective layer, which controls the gas separation properties, coated on a porous support that provides the mechanical strength.^4^ TFC membranes can provide separations with high fluxes combined with high mechanical resistance and at a lower cost (the selective layer being the most expensive part), which makes TFC membranes very attractive for industrial application.^4,5^ TFC membranes can be prepared through several routes, such as solution coating, interfacial or plasma polymerization, or chemical vapor deposition.^5,6^ Solution coating is the most popular technique, thanks to its simplicity and ease of scale up. In order to increase the selectivity instead and, therefore, the purity of the product, mixed-matrix membranes (MMM) can be employed. MMMs are based on a dispersion of fillers within a polymeric matrix; the fillers are chosen to provide high selectivity, while the polymer matrix ensures ease of processability. The combination will thus enhance the separation performance of the membrane.^7^

By combining the advantages of TFC and MMM, TFC_MMMs are promising candidates for gas separations thanks to their high selectivity and permeance. Wu et al.^8^ improved the separation performance of PEBAX by 55% by incorporating fullerenol and fabricating TFC. The presence of fullerenol also reduced the plasticization effect. Lee et al.^9^ developed TFC_MMM based on a poly(vinyl imidazole)-poly(oxyethylene methacrylate) matrix mixed with ZIF-8. The optimized TFC_MMM lead to a high CO_2_ permeance of 4500 GPU and CO_2_/N_2_ selectivity of 32. Ma et al.^10^ developed ultrathin TFC_MMMs containing poly(ether imide) mixed with 3D and 2D MOFs, such as UiO-66, MIL-101(Cr), 2D CuBDC and Zn_2_(bim)_4_. They all exhibited higher CO_2_ permeance and CO_2_/CH_4_ selectivity than those of neat poly(ether imide). TFC_MMM with 2D CuBDC presented the best separation performance with CO_2_ permeance and CO_2_/CH_4_ selectivity that is 3.3 and 2.2 times higher than that of the neat polymer, respectively, thanks to its 2D structure and its small pore size. The introduction of MOFs decreased as well the plasticization behavior in the membrane. Xie et al.^11^ managed to develop TFC_MMM based on polyactive matrix mixed with a core–shell MOF nanoparticle (NP), which consists of a porous metal–organic framework (MOF) core and a polymeric (polyethylene glycol (PEG)-based) shell. The resulting TFC_MMMs showed a better CO_2_ permeance (468 to 490 GPU) and CO_2_/N_2_ selectivity (25 to 45) compared to the self-standing MMM.

Zeolitic imidazolate frameworks (ZIFs) are one family of MOFs that has extensively been used in MMMs, particularly for CO_2_ separation from N_2_ in flue gases.^7,12−15^ They excel as fillers in membranes as they exhibit great structural and chemical tunability that can provide a wide range of selective adsorption sites.^1^ For instance, ZIF-94 has been widely employed in membranes for gas separations, because of the existence of 4-methyl-5-imidazolecarboxaldehyde (AmeIm) as a linker. The presence of aldehyde groups leads to a strong CO_2_ adsorption uptake (0.74 mmol g^–1^ at 298 K at 1 bar) and enhances the framework rigidity.^12^ In our previous study,^12^ self-standing MMMs containing Matrimid and ZIF-94 at different contents have been investigated. ZIF-94 shows good compatibility and good dispersion up to 20 wt %. The incorporation of ZIF-94 results in an increase in the CO_2_ permeability, explained by the increase in solubility due to the high CO_2_ capacity of ZIF-94. A concentration of 12 wt % was found to be optimal in order to get high CO_2_ permeability and CO_2_/N_2_ selectivity.

In this work, a solution of Matrimid mixed with ZIF-94 was used to produce TFC_MMM. Defect-free TFC_MMM containing Matrimid mixed with 12 wt % of ZIF-94 have been developed by using kiss coating, a variation of the dip coating technique,^16^ on a porous polyacrylonitrile (PAN) support. The TFC membranes have been characterized in terms of morphology by SEM analysis, of surface properties (water contact angle and FTIR) and by single gas permeation of N_2_, CH_4_, He, and CO_2_ from 1 to 10 bar. The TFC membranes were compared to the corresponding self-standing membranes. TFC_MMM presented the best results in terms of separation performance, with a higher permeance as expected but also higher CO_2_/N_2_ selectivity, and moreover they were stable at high pressure without suffering from any plasticization.

## 2 Material and methods

### 2.1 Materials

Matrimid 5218 was kindly provided by Hunstman (Switzerland). Polyacrylonitrile PAN support (pore size <50 nm) was purchased from Deltamen (Switzerland). ZIF-94 was kindly supplied by the University of St. Andrews (Prof. Paul Wright). It has been comprehensively characterized (CO_2_ and N_2_ uptake, particle size, heat of CO_2_ adsorption, BET and accessible volume) in our previous study.^12^ Dichloromethane (99.9%) was purchased from Acros Organics.

#### 2.1.1 Preparation of Self-Standing Membranes (12%wt_ZIF94)

Matrimid 5218 was dried overnight at 100 °C under vacuum. 0.5 g of dried polymer was dissolved in 20 mL of dichloromethane (1.5 wt %) and stirred for 1 h at room temperature. In the meantime, 0.07 g of ZIF-94 particles were added in CH_2_Cl_2_ (2 g) and ultrasonicated for 1 h. Then, the two solutions were combined, stirred overnight at ambient temperature, and sonicated for 10 min before casting. The resulting solution was poured into a 5 cm glass Petri dish. The membrane was allowed to form by slow solvent evaporation for 24–36 h in a fume cupboard.

As a reference, membranes based on the neat polymer were prepared by an identical procedure. The thicknesses of Matrimid and MMM were ∼45 and 50 μm, respectively, according to averaged measurements performed with a digital micrometer (Mitutoyo) at different locations on each membrane.

#### 2.1.2 Preparation of TFC Membranes (12%wt_ZIF94)

**Figure 1:**
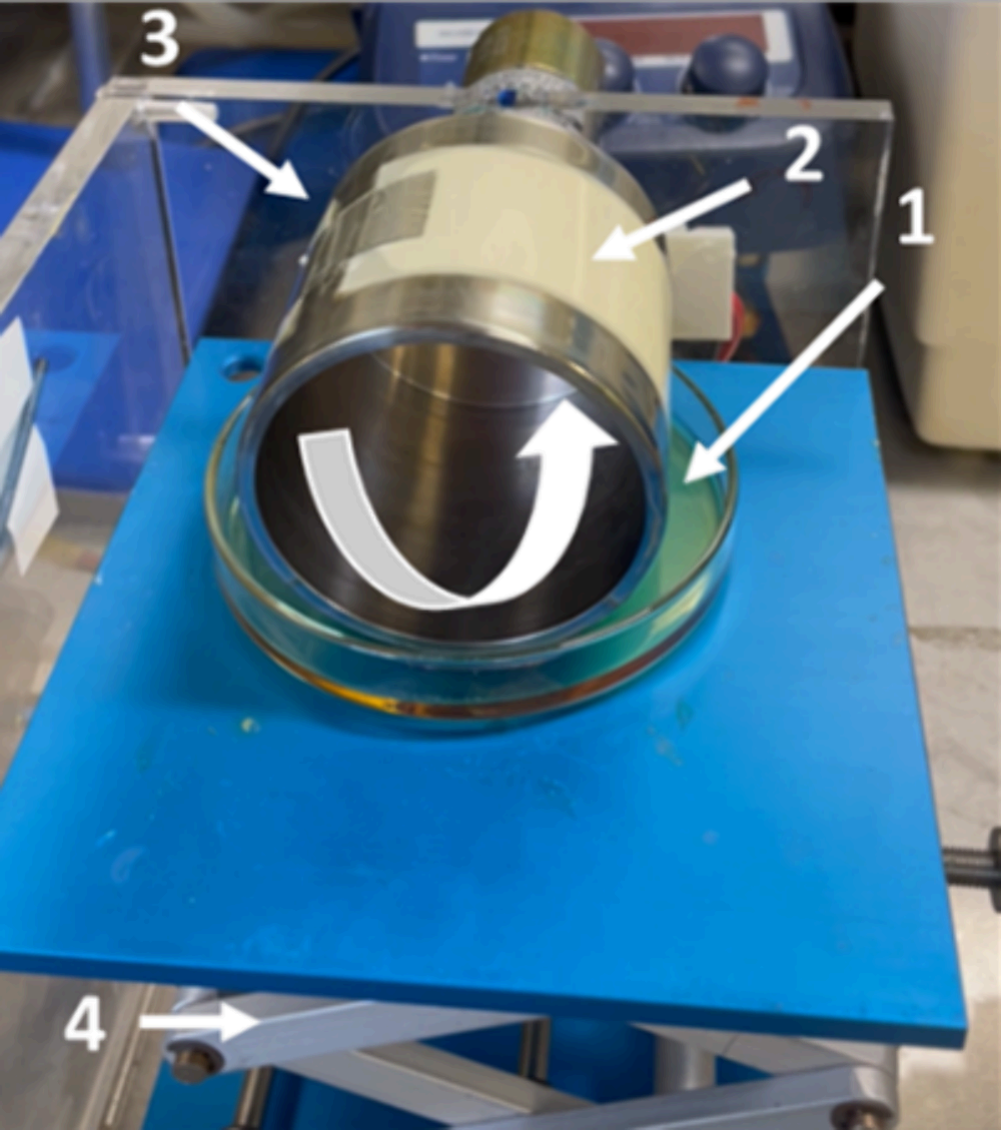
Roller coater system. Legend: (1) coating solution, (2) coated PAN, (3) tape, and (4) lab jack, which was used for adjusting the height.

TFC membranes were prepared using the kiss coating technique (Figure 1). Solutions of pure Matrimid or Matrimid_ZIF94 at 1.5 wt % in dichloromethane were coated on a PAN porous support. The same solutions as the ones used for self-standing membrane preparation were used.

For each coating, a sheet of 7 cm × 4 cm of PAN was attached to the roller wheel with aluminum tape resistant to solvent, and the support was put in contact with the solution by adjusting the height of the solution tray. When the PAN was in contact with the solution, the roller wheel was put in motion using a DC power supply. After two turns of the wheel, the solution tray was removed and the TFC was allowed to dry on the wheel for 2 h while spinning. When dried, the membranes were peeled off of the wheel and placed in a plastic bag. The thickness of the selective layer was determined by SEM.

### 2.2 Gas Permeance Testing

Single gas permeation measurements were carried out using a custom-built constant volume-variable pressure apparatus using pure N_2_, CH_4_, He and CO_2_ with pressures up to 15 bar at 25 °C.^17^

Membranes were first tested at 1 bar for the four gases, in the following order: N_2_, CH_4_, He, and CO_2_. After CO_2_, another test of N_2_ at 1 bar was completed in order to check any change in the membrane structure. Then, the pressure dependence was investigated for each gas using a set of run for N_2_ up to 10 bar, for CH_4_ up to 4 bar, for helium up to 4 bar and for CO_2_ up to 10 bar with increments of 1 bar. At the end of each set of gases, a test of N_2_ at 1 bar was performed to check any change in membrane structure. Between each increment of pressure and each set of measurement, the membrane was evacuated at least 30 min for TFC and 2 h for ST samples.

The permeation tests were performed on samples aged for 2–3 months. For each membrane, three different samples were tested, corresponding to three different locations on the membrane. The errors were calculated based on these three measurements.

### 2.3 SEM

The membranes were examined with a Model JSM-IT100 SEM system (JEOL, Japan) operating at 10 kV. Before SEM analysis, the samples were fractured in liquid nitrogen and then sputtered with a layer of 12 nm gold to form a conductive surface.

### 2.4 Contact Angle

Water contact angle (θ) measurements were taken with a mobile phone and analyzed with ImageJ using snake drop analysis.

The measurements were taken at room temperature using distilled water. The measurements were repeated three times on three different locations of the samples and averaged. The tangent method was used to calculate the contact angle. For each measurement, a 5 μL droplet was dispensed onto the samples.

### 2.5 FTIR

Fourier transform infrared spectroscopy (FTIR) of the membranes was performed on a VERTEX 70v (Bruker). Powder ZIF-94 was analyzed by using a diffusion cell (Praying Mantis, Harrick Scientific Products). Spectra of self-standing membranes were obtained by transmittance, whereas for the thin film composites they were obtained by ATR using a ZnSe crystal.

## 3 Results and Discussion

### 3.1 Characterization of TFC

**Figure 2:**
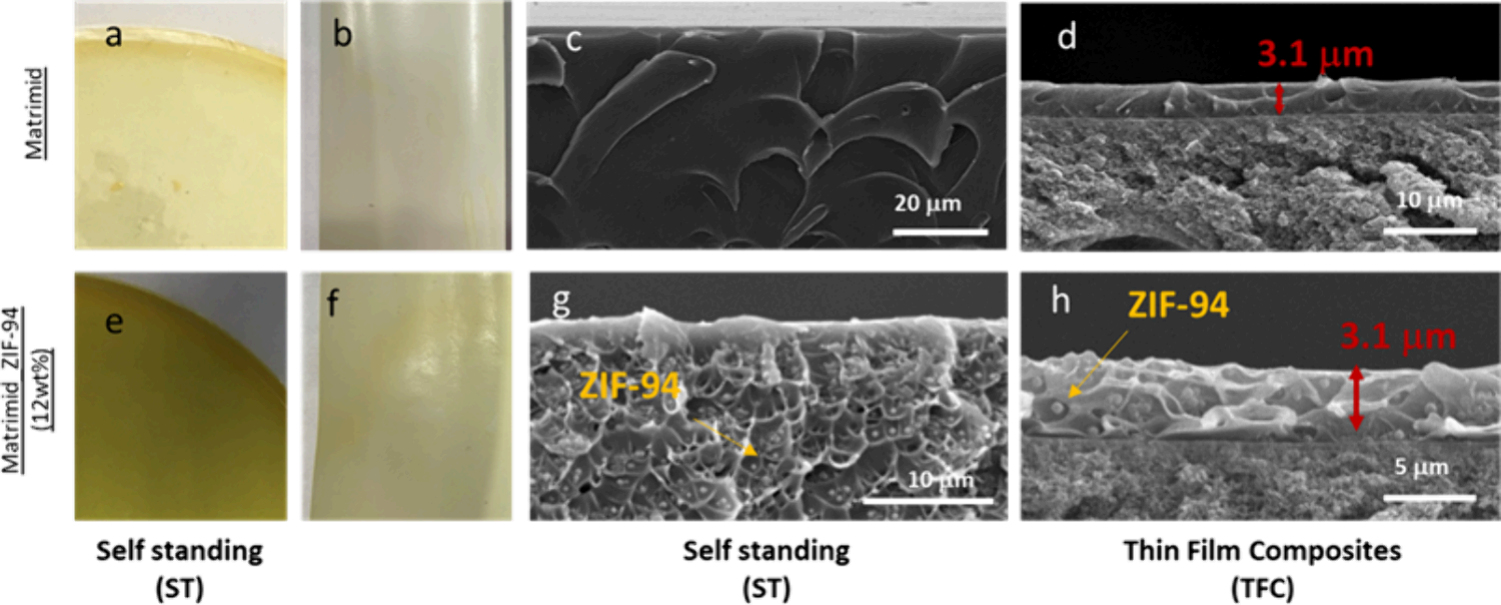
Photographs and cross sections SEM of self-standing and TFC membranes prepared from Matrimid solutions (top line, panels (a)–(d)) and Matrimid_ZIF-94 solutions (bottom line, panels (e)–(h)).

TFC were obtained by kiss coating using a solution of Matrimid and Matrimid/ZIF94 dissolved in dichloromethane. Photographs and cross-sectional SEM images of self-standing (ST) and TFC membranes are shown in Figure 2. The presence of ZIF-94 is evidenced by the change of color of the sample. The membrane becomes less transparent with ZIF-94 (see Figures 2e and 2f), for both membranes, ST and TFC. ZIF-94 has been also noticed on the SEM images (Figures 2g and 2h) with the presence of circular particles.

As shown in Figure 2d, Figure 2h, and Figure S1, TFC_Matrimid and TFC_MMM were obtained with a similar thickness of 3.1 μm. The selective layer on TFC should be minimized in order to get higher flux and higher permeances. TFC_MMM with a thinner selective layer, around 1 μm (Figure S2) has been also fabricated but unfortunately with defects. The ZIF-94 particle size^12^ is around 500 nm which is very large compared to the selective layer and leads to defects if the selective layer is too thin.

The water contact angles of the membrane surfaces (Figure S3) were measured to investigate the changes in the surface properties. The contact angle increases with the coating, as shown in Table S1. The neat PAN exhibits a hydrophilic character with a water contact angle of 51.5°, whereas the TFC membranes are hydrophobic with contact angles superior to 80°, due to the presence of Matrimid. The incorporation of ZIF-94 induced also an increase in the water contact angle, both for the ST and TFC membranes. This is due to the surface roughness brought by ZIF-94, as shown in Figure S4.

**Figure 3:**
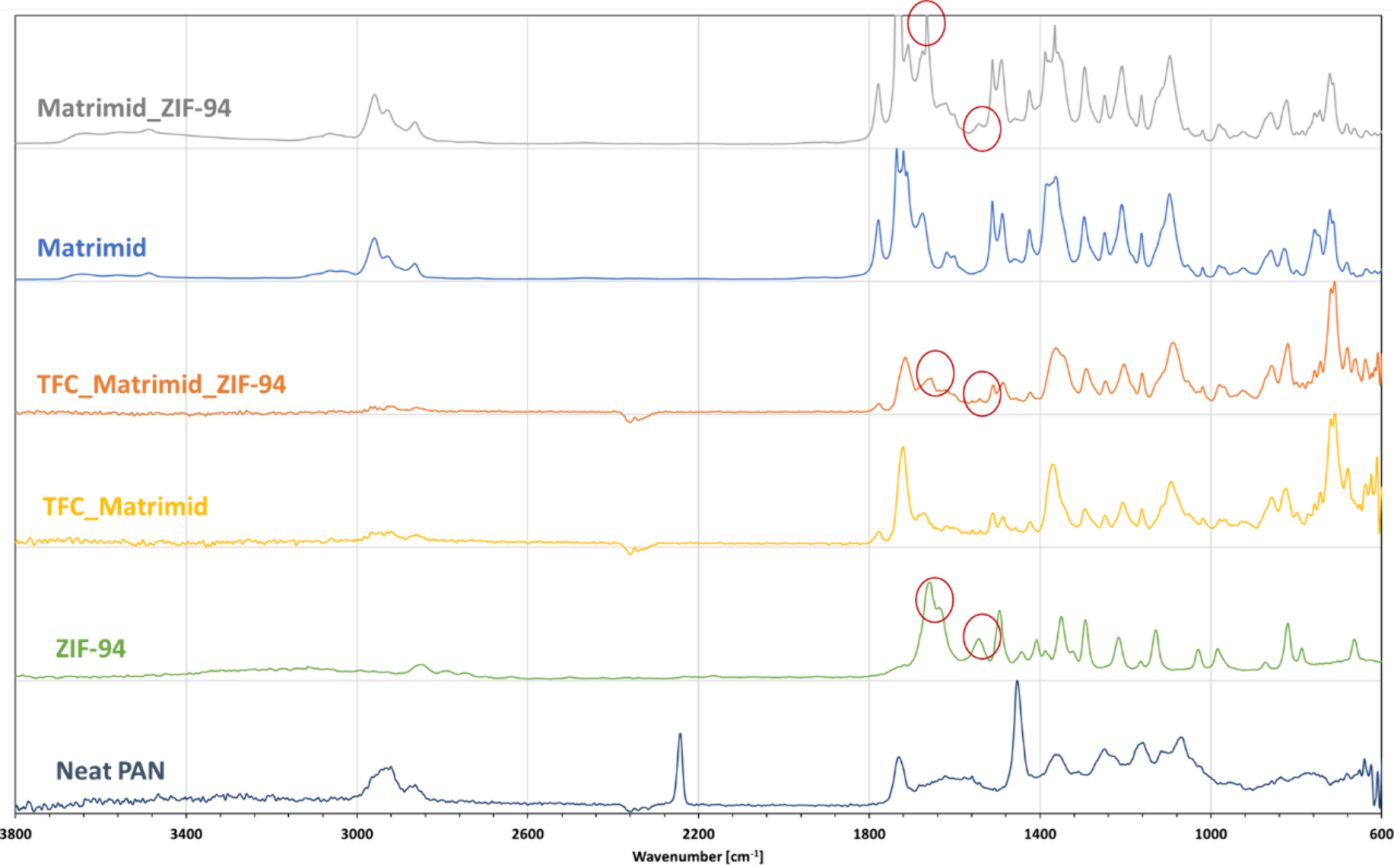
FTIR spectra of neat PAN, ZIF-94, TFC_Matrimid, TFC_Matrimid_ZIF-94, ST_Matrimid and ST_Matrimid-ZIF-94.

The infrared characterization of the membranes (ST and TFC) is shown in Figure 3. All of the characteristic peaks are listed in Table S2. Due to their similar chemical moieties, ZIF-94, Matrimid and PAN present similar spectra. However, the presence of ZIF-94 can be confirmed by the appearance of two new peaks at 1660 and 1540 cm^–1^ corresponding to the N–H bond. The full coverage of the PAN is also confirmed by the disappearance of the PAN characteristic peaks, such as 2220 cm^–1^ (nitrile group, CN) and 1450 cm^–1^ (methylene group, CH_2_).

### 3.2 Separation Performances

#### 3.2.1 1 bar

Pure gas permeances measurements of PAN, self-standing, and TFC membranes were carried out by using a constant pressure/variable volume apparatus at 25 °C and 1 bar. The permeances and selectivity are presented in Table 1.

**Table 1:** Pure Gas Permeances and Selectivity for PAN, ST, and TFC Membranes^a^

	Permeance^b^ [GPU]				Selectivity					
membrane^a^	N_2_	CH_4_	He	CO_2_	N_2_/CH_4_	He/N_2_	He/CH_4_	He/CO_2_	CO_2_/N_2_	CO_2_/CH_4_
PAN	7 × 10^4^ (±2%)	9 × 10^4^ (±2%)	17 × 10^4^ (±2%)	6 × 10^4^ (±2%)	0.8	2.4	1.9	2.9	0.8	0.6
ST_Matrimid (45 μm)	9 × 10^–3^ (±20%)	7 × 10^–3^ (±20%)	5 × 10^–1^ (±10%)	3 × 10^–1^ (±10%)	1.2	59	72	1.9	31	38
ST_MMM (50 μm)	1 × 10^–2^ (±20%)	2 × 10^–2^ (±20%)	5 × 10^–1^ (±10%)	4 × 10^–1^ (±10%)	0.6	55	34	1.5	36	23
TFC_Matrimid (3 μm)	1 × 10^–1^ (±10%)	1 × 10^–1^ (±10%)	6 (±2%)	3 (±2%)	1	53	57	1.8	29	31
TFC_MMM (3 μm)	1 × 10^–1^ (±10%)	2 × 10^–1^ (±10%)	8 (±2%)	4 (±2%)	0.6	80	45	2	40	22

a Value given in parentheses, “(X μm)”, is the thickness of the selective layer.

b Permeance = permeability/X.

The PAN permeation measurements of CH_4_, He, N_2_, and CO_2_ gases (Table 1) showed permeances inversely proportional to the square root of the molar masses of the gases (Figure S5), demonstrating that the mechanism of gas permeation through the porous supports is based on the Knudsen mechanism. The linear trend indicates the absence of a transport resistance and confirms that the selected support is suitable for the preparation of TFC membranes in which the porous support must not influence the gas transport properties.

In contrast, for ST and TFC membranes, the gas transport can be described by a solution-diffusion mechanism. The gas permeability coefficient (*P*) can be written as the product of a diffusivity coefficient (*D*) and a solubility coefficient (*S*) (see eq 1).
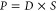
1

The ST and TFC membranes showed lower permeances but higher selectivities than PAN, which indicates the absence of defects in the selective layer.

Compared to ST membranes, TFC membranes presented a higher permeance for the four gases following expectations, since a thinner selective layer induces a lower mass-transfer resistance and, thus, a rise in permeance. The incorporation of ZIF-94 induced an increase in gas permeance for CH_4_, He, and CO_2_ gases as well. The improvement of CH_4_ and CO_2_ permeances can be explained by the high CO_2_ and CH_4_ adsorption capacity of ZIF-94.^18^ In our previous work,^12^ we showed that the addition of ZIF-94 inside a Matrimid matrix induced an increase in CO_2_ permeability due to a rise in the CO_2_ solubility resulting from the high CO_2_ sorption of ZIF-94, compared to the neat Matrimid. For the small gas (He), the increase in permeance was different between the TFC and ST membranes. In the latter, no variation was noticed, whereas in TFC membranes, He permeance increased by 35%. This increase in He permeance is more noticeable in TFC due to the higher polymer mobility in thin system, because a reduction in thickness induces a decrease in the glass-transition temperature (*T*_g_), which results in an increase in the mobility of the polymer chain and, by consequence, a higher permeance, as reported in the literature.^19,20^ As Matrimid is a glassy polymer, its gas permeability is correlated to its free volume fraction (FFV)^21^ and any factor that tends to modify its chain mobility will induce modification in FFV and, thus, gas permeability. As helium is small, it will be more affected by any chain movement.

For N_2_, the permeance remains actually stable in both kinds of membranes as the variation is consistent with the uncertainty of the measurements that is large due to the low permeability, which is similar to the leak of the permeation system.

**Figure 4:**
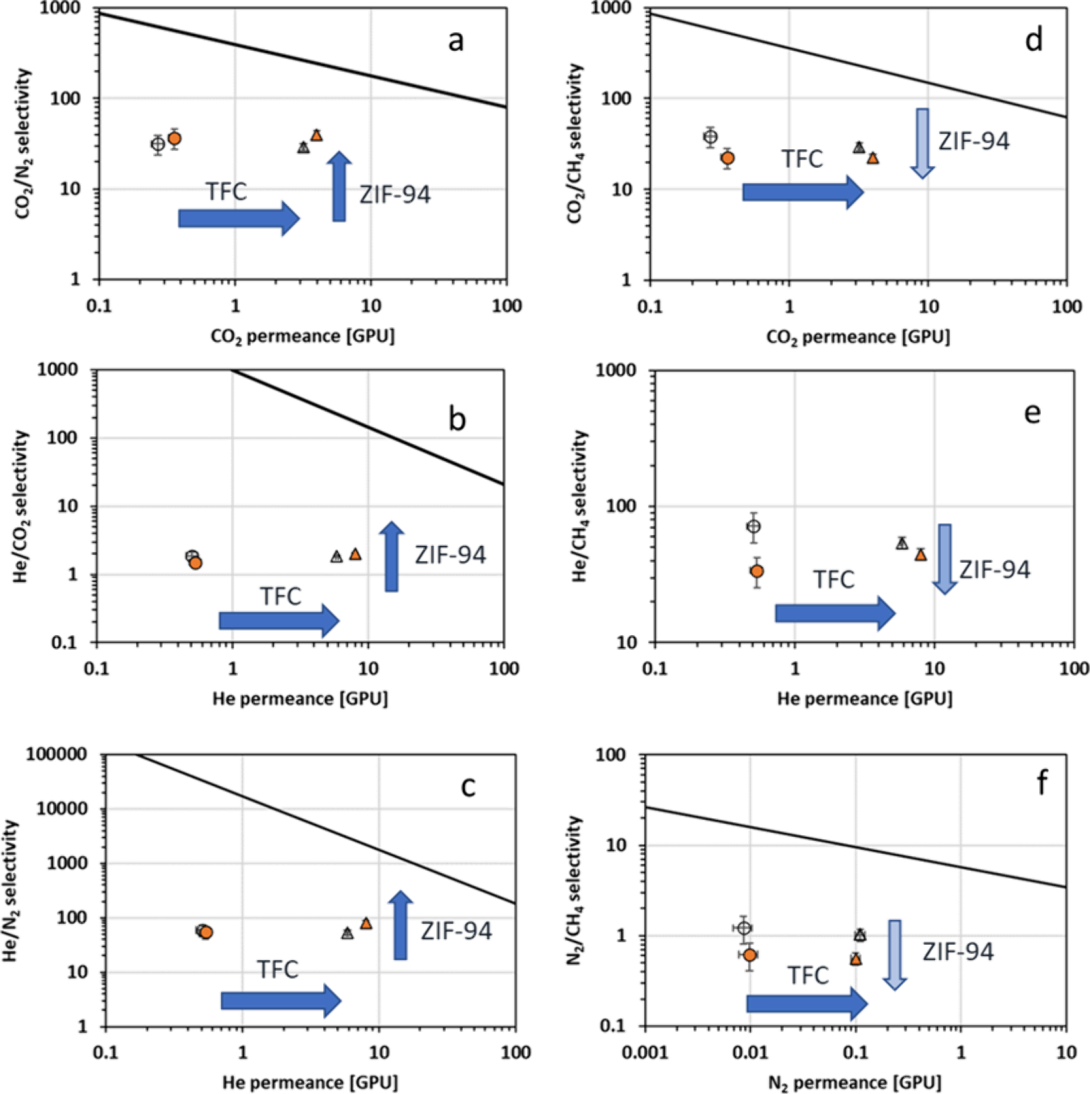
Membrane performance at 1 bar and 25 °C relative to the 2008 upper bound plot: (a) CO_2_/N_2_, (b) He/CO_2_, (c) He/N_2_, (d) CO_2_/CH_4_, (e) He/CH_4_, and (f) N_2_/CH_4_ (empty marker = Matrimid alone, filled marker = MMM, triangle = TFC membranes, circle = ST membranes). Note: The upper bound is assumed to be an effective membrane thickness of 1 μm.

The selectivity versus permeability (Robeson’s plots) for the gas pairs CO_2_/N_2_, He/CO_2_, He/N_2_, CO_2_/CH_4_, He/CH_4_, and N_2_/CH_4_ are shown along with the corresponding trade-off lines in Figure 4.

As shown in Figure 4, TFC membranes show a larger permeance for all the gases, thanks to the thinnest selective layer. The incorporation of ZIF-94 has a positive impact for the He/CO_2_, CO_2_/N_2_ and He/N_2_ separation with a shift toward larger selectivity.

**Figure 5:**
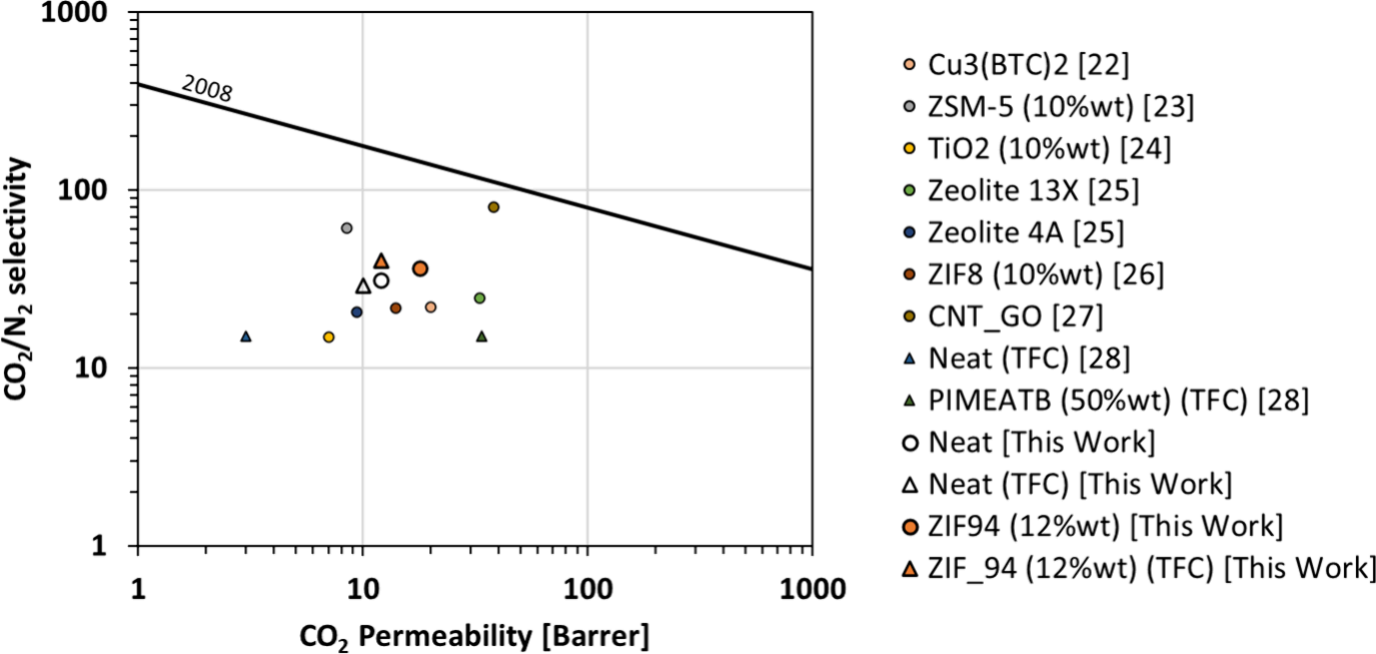
Separation performances for CO_2_/N_2_ for several composite Matrimid-based membranes.^22,23,24,25,26,27,28^ (Circle markers represent self-standing membranes, and triangle markers are for TFC membranes.)

In terms of the CO_2_/N_2_ separation (Figure 5), TFC based on Matrimid mixed with ZIF-94 presents higher selectivity than other Matrimid-based MMMs. Note that the CO_2_/N_2_ selectivity of TFC_MMM is 2 times higher than the minimum required for an industrial application (i.e., 20 (ref (3))). CO_2_ permeances are still quite low, compared to what it is expected for an industrial application (i.e., 1000 GPU (ref (3))); however, this could be improved by using smaller filler particles and decreasing the thickness of the selective layer.

#### 3.2.2 Pressure Dependence

**Figure 6:**
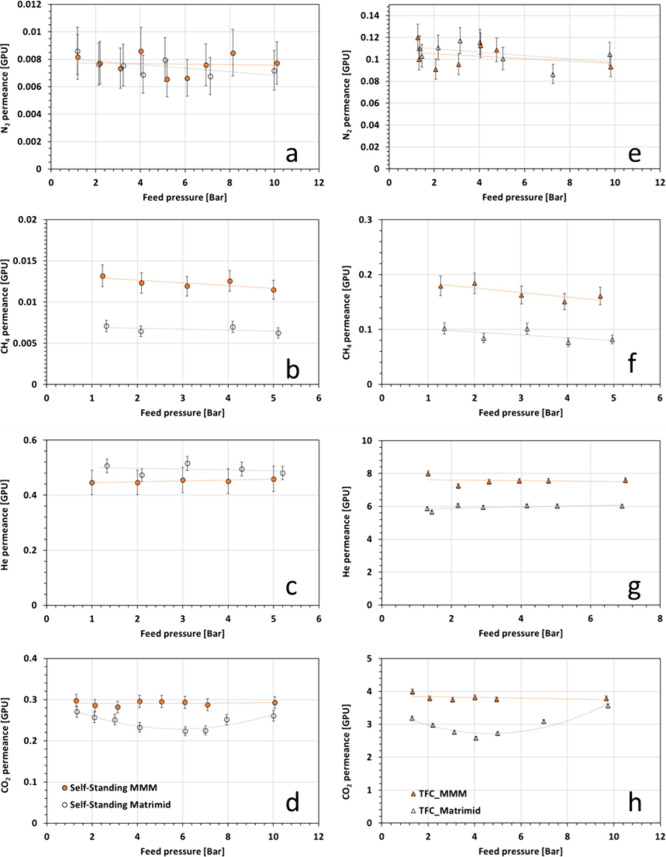
Pressure dependence for self-standing membranes (panels (a)–(d); circle symbols are used as data markers) and TFC membranes (panels (e)–(h); triangle symbols are used are data markers) of N_2_, CH_4_, He, and CO_2_ permeance (empty symbols = Matrimid alone, filled symbols = MMM).

The variation of permeance for N_2_, CH_4_, He, and CO_2_ for self-standing and TFC membranes with the feed pressure is shown in Figure 6.

For N_2_, the permeance change with increasing feed pressure was within the range of experimental error. For CH_4_, the permeance slightly decreased with increasing feed pressure with a decrease of 12% for ST _Matrimid, 14% for ST_MMM, 20% for TFC_Matrimid, and 16% for TFC_MMM. For the smaller gas, i.e., helium, the permeance was stable over the range of pressure both for TFC and self-standing membranes. This is in agreement with the dual-sorption model for glassy polymers, which predicts a slight decrease in permeability with increasing pressure for N_2_, CH_4_, and He, due to the saturation of Langmuir sites and the compaction of polymer matrix. This also suggests that the pressure dependence has a larger impact on the larger gases. Smaller gases such as He are less impacted by the reduction of the FFV.

Concerning CO_2_ experiments, a different behavior emerges for the membranes without ZIF-94 (see Figures 6d and 6h) with the presence of a minimum in CO_2_ permeance at 7 bar for ST membrane and at 4 bar for TFC membrane, respectively. This pressure corresponds to the plasticization pressure. Glassy polymers such as Matrimid are inclined to suffer from plasticization, where sorbing penetrant such as CO_2_ modifies the interactions between adjacent segments of polymer chains, leading to a swelling of the polymer matrix and an increase in gas permeability. The plasticization pressure in ST membrane is higher than in TFC membrane due to the thickness, as reported in the literature.^29−32^ TFC are more sensitive to plasticization than thick films as polymer chains are more flexible within thin film, so more sensitive to interaction with gases.

In contrast, for MMMs, ST_MMM and TFC_MMM, the CO_2_ permeance was stable over the range of pressure, with a variation of 5% for ST_MMM and 1% for TFC_MMM. No plasticization pressure was noticed. The incorporation of ZIF-94 suppresses the plasticization effect in the range of the experimental pressure, i.e., up to 10 bar. The filler acts as a rigidifying agent, which constrains the polymer chain and avoids the swelling.^7,12^

**Figure 7:**
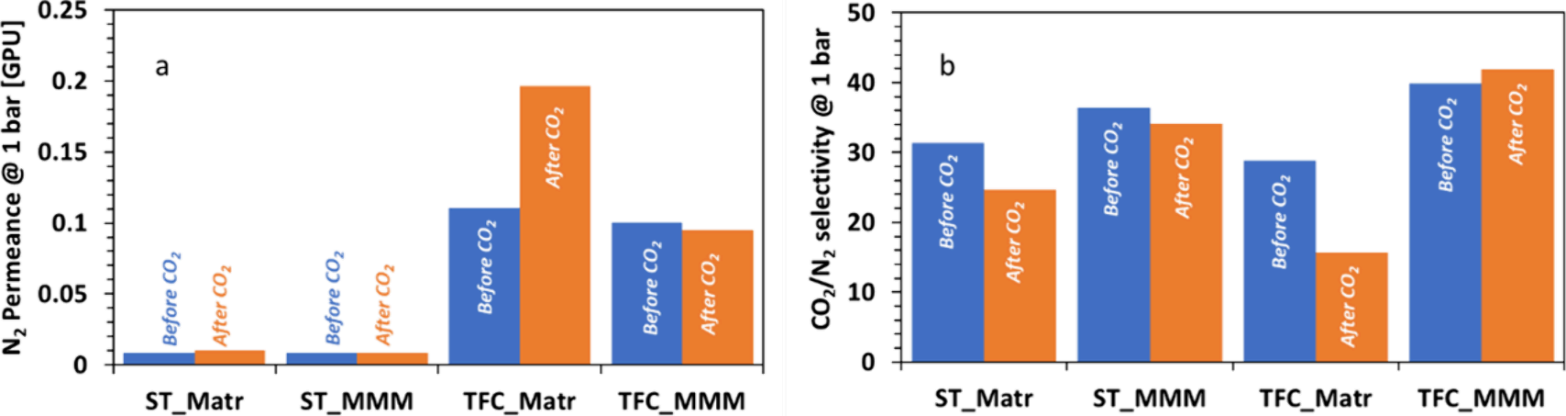
(a) N_2_ permeance and (b) CO_2_/N_2_ selectivity at 1 bar before and after being in contact with CO_2_. (Blue color represents data before CO_2_; orange color represents data after CO_2_.)

Plasticization can also induce irreversible changes and this phenomenon is called the conditioning effect,^29^ where, after being in contact with CO_2_, polymer chains might not return to its initial structure. To investigate this conditioning effect, we measured N_2_ permeance at 1 bar before and after being in contact with CO_2_ at 10 bar. As shown in Figure 7a, N_2_ permeance did not change for the MMMs (variation of <5%), whereas for the membranes without filler, it exhibited an increase of 20% and 78% for ST and TFC, respectively. Within MMMs, no variations have been noticed, due to the fact that the membranes did not suffer any plasticization up to 10 bar.

For neat Matrimid, the TFC is more sensitive to the CO_2_ conditioning with a larger increase in N_2_ permeance. Horn et al.^29^ found similar results with thin film not returning to its initial permeance, whereas thick film exhibited reversibility after being exposed to CO_2_.

This conditioning effect is particularly important for industrial applications where mixed gases are usually used. As noticed above, the presence of CO_2_ induced plasticization and a nonreversible swelling in the membrane, which impacts the N_2_ permeance and by consequence the CO_2_/N_2_ selectivity. As shown in Figure 7b, after being in contact with CO_2_, the CO_2_/N_2_ selectivity declined for ST_Matrimid and TFC_Matrimid membranes, by 21% and 35%, respectively. The large decrease will have a negative influence on the membrane performance reducing the industrial applicability of TFC_Matrimid. However, as shown in Figure 7b, by incorporating ZIF-94, the CO_2_/N_2_ selectivity remains stable, for ST_MMM and TFC_MMM. This result showed the potentiality of using TFC_MMM for industrial applications, thanks to its high selectivity and its stability in the presence of CO_2_.

## 4 Conclusion

TFC membranes containing Matrimid mixed with 12 wt % ZIF94 have been produced on a porous PAN support for efficient CO_2_ separation by using a roller coater. Hydrophobic defect-free membranes with a selective layer of 3 μm have been obtained. In comparison to the self-standing membranes, the TFC exhibited higher CO_2_ permeance (4 GPU) and higher CO_2_/N_2_ selectivity (40). The incorporation of ZIF-94 resulted in an increase in CO_2_ and CH_4_ permeances within both membranes, thanks to the high CO_2_ and CH_4_ adsorption capacity of the filler. ZIF-94 had also a positive impact for the He/CO_2_, CO_2_/N_2_ and He/N_2_ separation with a shift toward the larger selectivity. The introduction of ZIF-94 also suppressed the plasticization effect. While the TFC and ST_Matrimid membranes presented a minimum in CO_2_ permeance, at 4 and 7 bar, respectively, the ST and TFC_MMM membranes showed a stable CO_2_ permeance over the range of pressure. The incorporation of ZIF-94 in a thin film composite not only improved the separation performances but also led to a better stability of the membranes at high pressure.

This investigation showed the potentiality of using TFC_MMM for a post-combustion application, thanks to its CO_2_/N_2_ selectivity of 40, which is twice as high as the minimum required for an industrial application.

## Supporting Information Available

Gas permeance of PAN; surface and cross-sectional SEM micrographs of TFC_MMM, water droplet on PAN, ST_Matrimid, ST_MMM, TFC_Matrimid, and TFC_MMM; water contact angle for PAN, ST_Matrimid, ST_MMM, TFC_Matrimid, and TFC_MMM; FTIR group frequencies for Matrimid, ZIF94 and PAN; SEM micrographs of the surface of PAN and TFC (PDF)
