# Supplementary material for: Thin Film Composite Mixed-Matrix Membranes Based on Matrimid and Zeolitic Imidazolate Frameworks for CO2/N2 Separation Performance
Source: Ind Eng Chem Res. 2024 Nov 11;63(46):20356–64. doi: 10.1021/acs.iecr.4c03086 (PMC11583212; doi:10.1021/acs.iecr.4c03086)
Supplement: Supplementary file 1 — ie4c03086_si_001.pdf [file ie4c03086_si_001.pdf]

Supplementary Information

Thin Film Composite Mixed-Matrix Membranes based on Matrimid and Zeolitic Imidazolate Frameworks for CO_2/N_2 separation performance

Elsa Lasseuguette[1,*], Maria-Chiara Ferrari[1]

[1] : School of Engineering, University of Edinburgh, Robert Stevenson Rd, Edinburgh EH9 3FB, U.K.

[*]: Corresponding author: e.lasseuguette@ed.ac.uk

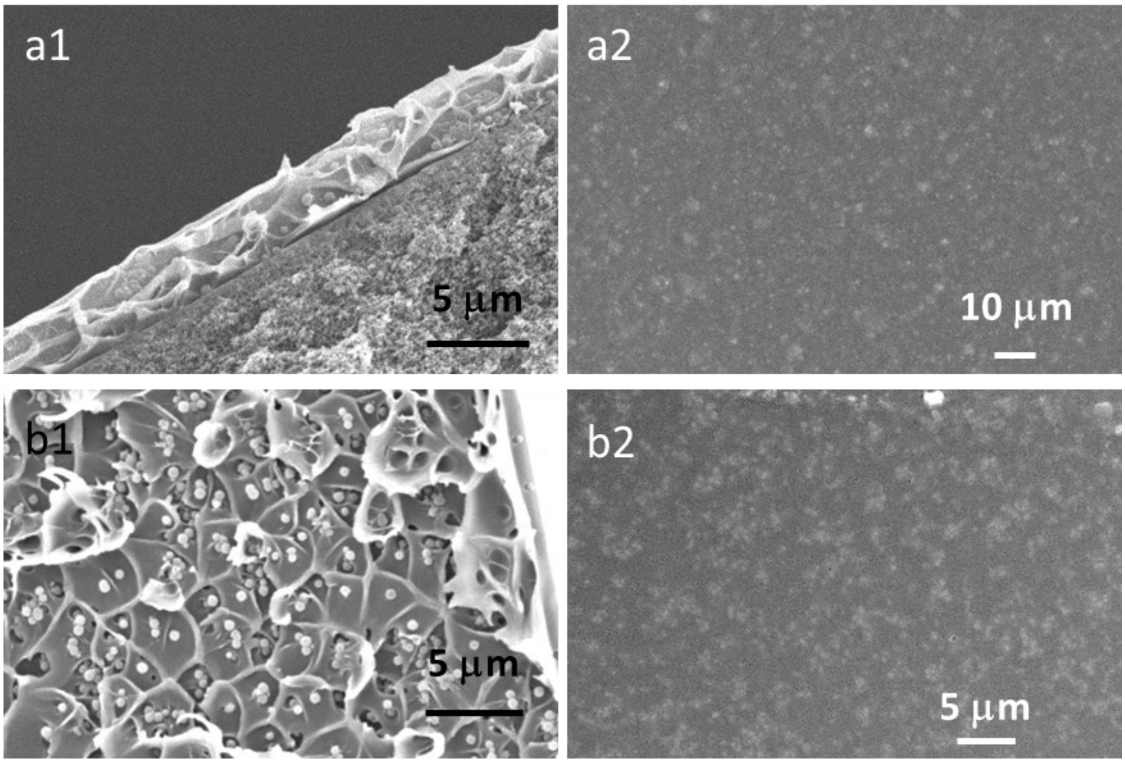

Figure S1: SEM images of TFC_MMM (a) (Cross-section a1 and Surface a2) and MMM (b) (Cross-section b1 and Surface b2) at higher magnification.

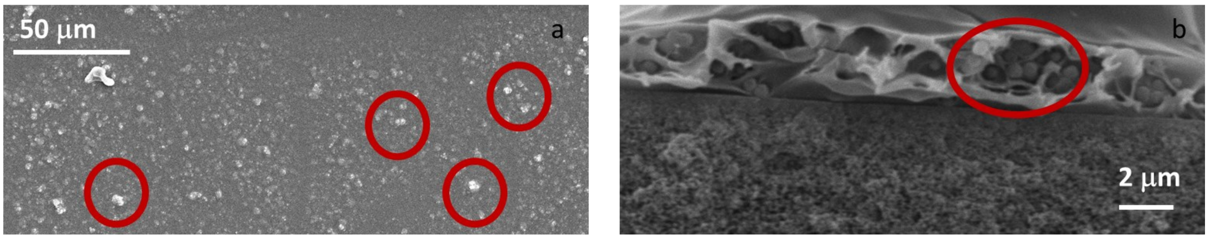

Figure S2: Surface(a) and cross section (b) SEM of TFC_MMM with selective layer of 1 µm. Red = defects.

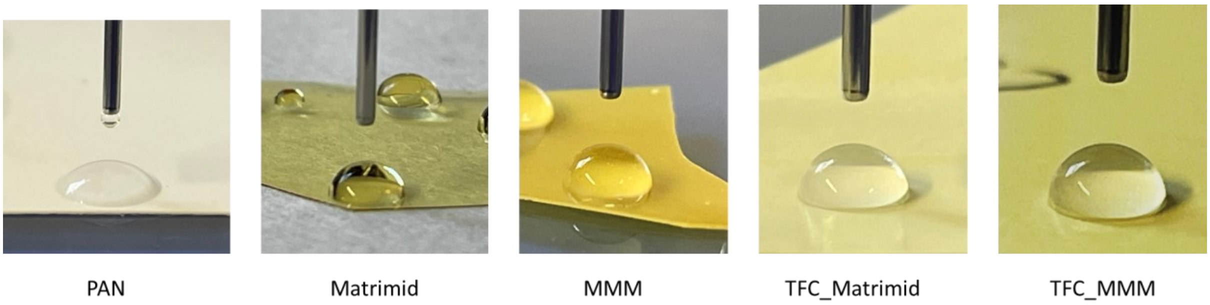

Figure S3: Water droplet on PAN, ST_Matrimid, ST_MMM, TFC_Matrimid and TFC_MMM.

PAN	ST_Matrimid	ST_MMM	TFC_Matrimid	TFC_MMM
51.5 ±1	81.5 ±2	91.7 ±3	82 ±2	93.2 ±3

Data obtained from Figure SI-3 analysed by ImageJ with DropSnake analysis

Table S1: Water contact angle [°] for PAN, ST_Matrimid, ST_MMM, TFC_Matrimid and TFC_MMM (error determined on measurements).

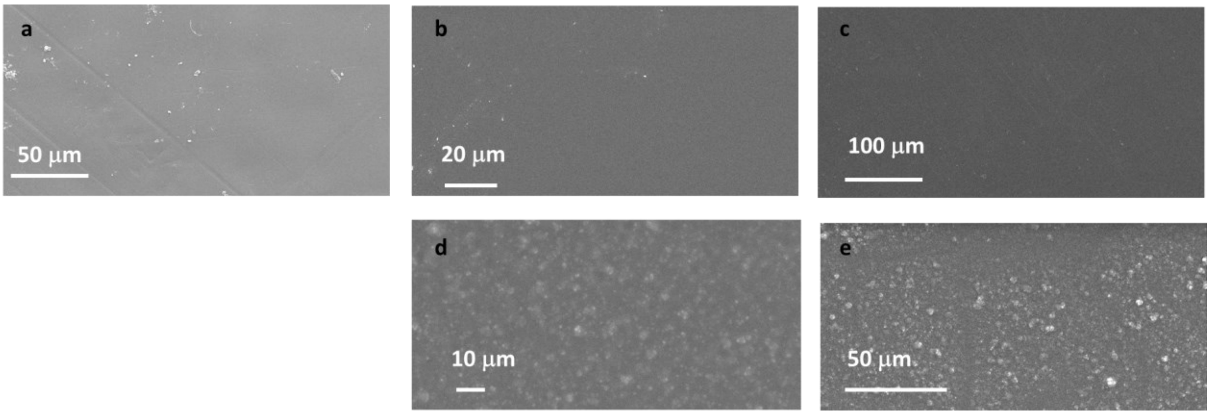

Figure S4: SEM surface of PAN (a), Self-Standing and TFC Membranes prepared from Matrimid (Top line, b, c) and Matrimid_ZIF-94 (Bottom line, d,e) solutions.

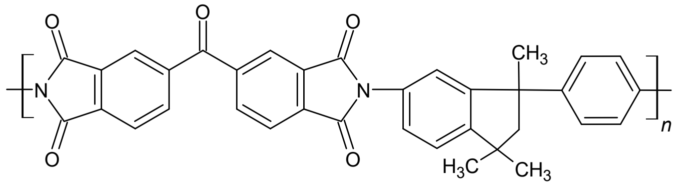 **Matrimid**	
CH	*3060-2870/925-720 cm^{-1}*
C-C=O	*1775/1670/1200 cm^{-1}*
C=C	*1615/1500 cm^{-1}*
C-N-C	*1295-1260 cm^{-1}*
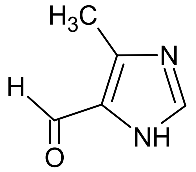 **ZIF-94**	
NH	*1660/1540 cm^{-1}*
C=N	*1130 cm^{-1}*
C=0	*1605 cm^{-1}*
C-N-C	*1295-1260 cm^{-1}*
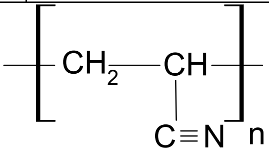 **PAN**	
C≡N	*2200 cm^{-1}*
CH_2	*2930-2870/1460-1450/1380-1360 cm^{-1}*

Table S2: FTIR group frequencies for Matrimid, ZIF94 and PAN.

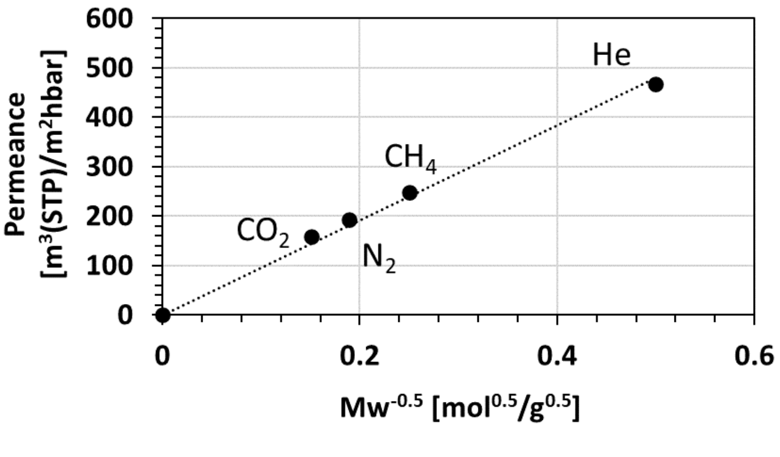

Knudsen diffusion: $Perm \propto d_{Pore}\sqrt{\frac{RT}{3\,M_{gas}}}$

Figure S5: Gas Permeance (N_2, CH_4, He, CO_2) of PAN function of Molecular weight of each gas. (Filled marker: experimental data, dashed line: Knudsen diffusion law).
